# The Polarization of the G-Protein Activated Potassium Channel GIRK5 to the Vegetal Pole of *Xenopus laevis* Oocytes Is Driven by a Di-Leucine Motif

**DOI:** 10.1371/journal.pone.0064096

**Published:** 2013-05-22

**Authors:** Beatriz Díaz-Bello, Claudia I. Rangel-García, Carolina Salvador, Rolando Carrisoza-Gaytán, Laura I. Escobar

**Affiliations:** Departamento de Fisiología, Facultad de Medicina, Universidad Nacional Autónoma de México, Ciudad de México, México; Virginia Commonwealth University, United States of America

## Abstract

The G protein-coupled inwardly-rectifying potassium channels (known as GIRK or Kir3) form functional heterotetramers gated by G-βγ subunits. GIRK channels participate in heart rate modulation and neuronal postsynaptic inhibition in mammals. In *Xenopus laevis* oocytes, GIRK5 is a functional homomultimer. Previously, we found that phosphorylation of a tyrosine (Y16) at its N-terminus downregulates the surface expression of GIRK5. In this work, we elucidated the subcellular localization and trafficking of GIRK5 in oocytes. Several EGFP-GIRK5 chimeras were produced and an ECFP construct was used to identify the endoplasmic reticulum (ER). Whereas GIRK5-WT was retained in the ER at the animal pole, the phospho-null GIRK5-Y16A was localized to the vegetal pole. Interestingly, a construct with an N-terminal Δ25 deletion produced an even distribution of the channel in the whole oocyte. Through an alanine-scan, we identified an acidic cluster/di-leucine sorting-signal recognition motif between E17 and I22. We quantified the effect of each amino acid residue within this di-leucine motif in determining the distribution of GIRK5 to the animal and vegetal poles. We found that Y16 and I22 contributed to functional expression and were dominant in the polarization of GIRK5. We thus conclude that the N-terminal acidic di-leucine motif of GIRK5 determines its retention and polarized trafficking within Xl oocytes.

## Introduction

*Xenopus laevis* (*Xl*) oocytes are the best characterized model for maturation and fertilization. Like other oocytes, XI oocytes are asymmetric cells with a complex internal polarity. On one side, the dark animal pole contains the nucleus, the endoplasmic reticulum (ER) and pigmented granules [1]; on the other side, the germ plasm and a mitochondrial cloud are distributed evenly in the cytoplasm towards the yellow pale vegetal pole [2], [3]. In oocytes, the asymmetry of mRNAs contributes to the maintenance and organization of the cytokeratin network in the vegetal cortex [4], [5]. Despite the well characterized asymmetry of mRNAs and subcellular organelles in oocytes, little is known about the polarization of their maternal proteins.

**Figure 1:**
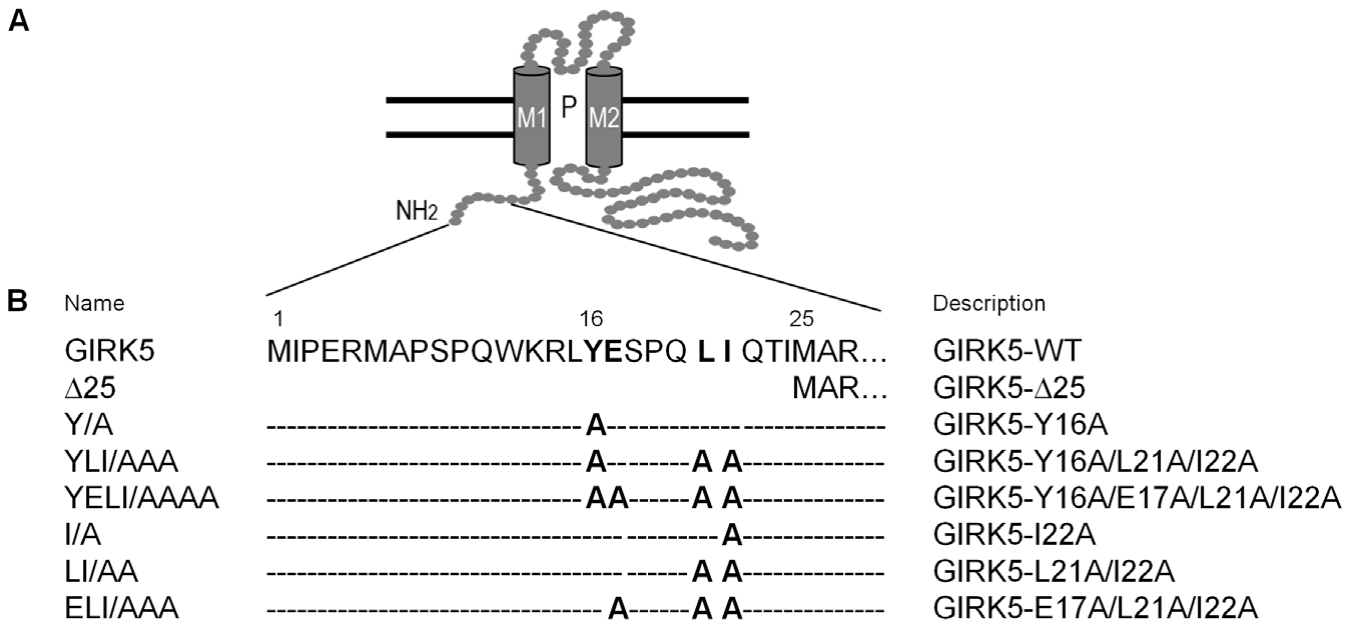
Cytoplasmic N-terminal domain of a GIRK5 subunit and constructs generated. A) GIRK5 is composed of a short intracellular N-terminus, two trans-membrane helices (M1 and M2), a pore (P), an extracellular loop, and a long unstructured C-terminus (Bichet D., 2003 and Choe S., 2002). The N-terminus of GIRK5 contains an acidic di-leucine motif (16-YEXXXLI-22) that drives its cellular trafficking. B) Name and description of each mutant used in this study.

G-protein regulated inward-rectifier potassium channels (GIRK) are a subfamily of proteins that participate in signaling responses at inhibitory synapses in mammals [6], [7]. Up to now, four members of this family, known as GIRK1-4 or Kir3.1-3.4, have been identified in mammals. These channels are either homotetramers (GIRK2) or heterotetramers of GIRK1 that are activated by G-βγ subunits. They are present in non-excitable cells such as platelets [8], lung cancer [9] and breast cancer [10], [11]. Another member of this family is GIRK5, an endogenous G-protein activated potassium channel present only in *Xl* oocytes [12]. GIRK5 is a homotetramer that helps to maintain a hyperpolarized resting membrane potential; GIRK5 shows a basal activity due to an endogenous G-βγ protein pool that keeps oocytes meiosis arrest [13]–[16]. Interestingly, in contrast to its mammalian homologues, GIRK5 contains a particularly long N-terminus (Fig. 1).

Previously, we found that within this N-terminus, the dephosphorylation of Y16 determines whether GIRK5 is transported to the plasma membrane [15]. Since GIRK5 surface expression occurred only one hour after incubation with a Protein Tyrosine Kinase inhibitor, we hypothesized then that GIRK5 was retained in a subcellular compartment. In this report, we have determined the subcellular localization of GIRK5. We discovered that it is polarized to the vegetal pole, and that such polarization is dependent on a di-leucine motif (YEXXXLI) at its N-terminus. There is precedence of di-leucine motifs in contributing to the polarization of proteins. For example, several proteins are targeted to dendrites in neurons and basolateral membranes in epithelial cells in a di-leucine dependent manner [17]–[22], and several proteins depend on such motifs for trafficking at the level of the trans-Golgi network, endosomes, plasma membrane, and lysosomes [23]. However, this is the first study in which the role of di-leucine motifs in polarizing maternal ion channels like GIRK5 is investigated. Our study represents an important step forward in understanding how the localization of maternal ion channels is carefully controlled in *Xl* oocytes.

## Materials and Methods

### DNA Constructs

Site-specific mutagenesis of Y16A, E17A, S18A, P19A, Q20A, L21A and I22A was performed by PCR, using GIRK5 cDNA (GenBank ID: AAB53154.1) as template and the primers listed in Table 1. In all cases, SP6 (sense) and Low 2 (anti-sense) were used as flanking primers. Constructs encoding enhanced green fluorescent protein (EGFP) fusion proteins were generated by adding the Open Reading Frame (ORF) of pEGFPC1 (Clontech) to the N-terminus of GIRK5 cDNA. All GIRK5 cDNAs and EGFP chimera constructs were subcloned into the pRSSP6013A3-UWE vector (pBF) and sequenced. The ORF of the ER marker, pECFP-ER (BD Living Colors, Clontech), was subcloned into the pRSSP6013A3-UWE vector (pBF) and sequenced. A summary of all the constructs used in this study is shown in Table 1.

**Table 1:** Primers used for site-specific mutagenesis.

Construct generated	Primer sequence
SP6	^5′^ - GATTTAGGTGACACTATAGAA - ^3′^
Low 2	^5′^ - AGA GAC CAA AAA GAG ACG ATC GTCGCC TGT ATC AAA - ^3′^
GIRK5-Y16A	^5′^ - AAAGATTGGCTGAGTCACC - ^3′^ (sense)
	^5′^ - GGTGACTCAGCCAATCTTT - ^3′^ (anti-sense)
GIRK5-E17A	^5′^ - GATTGTATGCGTCACCAC - ^3′^(sense)
	^5′^ - GTGGTGACGCATACAATC - ^3′^ (anti-sense)
GIRK5-S18A	^5′^ - TTGTATGAGGCGCCACAACTC - ^3′^ (sense)
	^5′^ - GAGTTGTGGCGCCTCATACAA - ^3′^ (anti-sense)
GIRK5-P19A	^5′^ - GATTGAGTCAGCACAACTCATC - ^3′^ (sense)
	^5′^ - GATGAGTTGTGCTGACTCATAC - ^3′^ (anti-sense)
GIRK5-Q20A	^5′^ - GAGTCACCAGCGCTGATCCAA - ^3′^ (sense)
	^5′^ - TTGGATGAGCGCTGGTGACTC - ^3′^ (anti-sense)
GIRK5-L21A	^5′^ - CACCACAAGCCATCCAAACC - ^3′^ (sense)
	^5′^ - GGTTTGGATGGCTTGTGGTG - ^3′^ (anti-sense)
GIRK5-I22A	^5′^ - CCACAACTCGCCCAAACCATC - ^3′^ (sense)
	^5′^ - GATGGTTTGGGCGAGTTGTGG - ^3′^ (anti-sense)
GIRK5-LI/AA	^5′^ - GAGTCACCACAAGCCGCCCAAACCATCATCGC - ^3′^ (sense)
	^5′^ - GCCATGATGGTTTGGGCGGCTTGTGGTGACTC - ^3′^ (anti-sense)
GIRK5-ELI/AAA	^5′^ - GATTGTATGCGTCACCAC - ^3′^ (sense)
(GIRK5-LI/AA as template)	^5′^ - GTGGTGACGCATACAATC - ^3′^ (anti-sense)
GIRK5-YI/AA	^5′^ - AAAGATTG GCT GAGTCACC - ^3′^ (sense)
(GIRK5-I/A as template)	^5′^ - GGTGACTCA GCC AATCTTT - ^3′^ (anti-sense)
GIRK5-YLI/AAA	^5′^ - GAGTCACCACAAGCCGCCCAAACCATCATGGC - ^3′^ (sense)
(GIRK5-Y/A as template)	^5′^ - GCCATGATGGTTTGGGCGGCTTGTGGTGACTC - ^3′^ (anti-sense)
GIRK5-YELI/AAAA	^5′^ - AAGATTGGCTGCGTCACC - ^3′^ (sense)
(GIRK5-LI/AA as template)	^5′^ - GGTGACGCAGCCAATCTT - ^3′^ (anti-sense)

### mRNA Synthesis and Microinjection

GIRK5, GIRK5 mutants, EGFP chimera and ECFP-ER mRNAs were synthesized from linearized plasmid vectors using the mMessage mMachine kit (Ambion Corporation). For microinjection, we used full-grown *(*stage VI) *Xl* oocytes, the needle was inserted in the vegetal hemisphere close to the equator, at an approximately 45-degree angle. Frogs were anesthetized and oocytes were obtained as described in [14]. Each oocyte was injected with 20 ng (50 nL) of mRNA. Control oocytes were injected with water. Oocytes were maintained in a ND96 solution (NaCl 96 mM, KCl_2_ mM, CaCl_2_ 1.8 mM, MgCl_2_ 1 mM, HEPES 5 mM, sodium pyruvate 2.5 mM; pH 7.4) at 18°C.

### Confocal Microscopy

Four days after mRNA injection oocytes were fixed with 2% paraformaldehyde (PFA; Sigma) in ND96 (to avoid nucleus deformation) for 10 minutes. Oocytes were incubated in 30% sucrose, 2% PFA, ND96 and kept at 4°C. Serial 10 µm slices were cryostat-cut (Leica CM1100) at −20°C, mounted on gelatin-coated slides, and cover-slipped with Vectashield mounting medium (Vector laboratories, CA, USA). EGFP and ECFP fluorescence was excited with an argon laser beam at 488 and 458 nm, respectively. A Leica TCSPS5 microscope was used. EGFP and ECFP emitted fluorescent light was collected between 500 and 544 nm, and between 473 nm and 524 nm, respectively. ECFP fluorescence was collected in blue and changed to red to facilitate the merge. To avoid crosstalk between the two channels, double-labeled images were acquired subsequently and emission was collected between 496–530 nm for EGFP and between 460–495 nm for ECFP. Images were taken using a 10× and 40× dry lens at the mid-section of each oocyte. The pixel density was 4.36×10^5^ pix/mm^2^ with a resolution of 1024×1024 pixels. Quantification of fluorescence was estimated with the Leica Application Suite, Advanced Fluorescence Lite 2.6.0 software. Comparison of the ion channel distribution between the animal and the vegetal pole were performed from independent regions-of-interest (ROI). The relative intracellular fluorescence was given as the ratio between one ROI and the whole-cell fluorescence intensity. Non-injected oocytes were used as a control of autofluorescence; the fluorescence below 50 gray values (autofluorescence) was subtracted from the injected oocytes.

### Western Blotting

The total membrane fraction was obtained by homogenization using 2 µl of lysis buffer (sucrose 250 mM, EDTA 1 mM, TRIS 1 mM, protease inhibitor mini Complete from Roche; pH 7.6) per oocyte, followed by two rounds of centrifugation at 200 g for 10 min at 4°C. The supernatant was mixed with 2 µl of Laemmli buffer per oocyte, heated to 95°C following the procedure described before [15].

### Electrophysiology

Whole-cell currents were recorded two to four days after mRNA injection with a twoelectrode voltage clamp amplifier (Geneclamp 500) and a Digidata 1322A interface with pClamp8 software (Axon Instruments) at room temperature (22–24°C), as described previously [15]. The recording solution contained: 118 mM KCl, 1 mM CaCl2, 2 mM MgCl2, and 5 mM HEPES, pH 7.4. The recording pipette was filled with 3 M KCl. Voltage pulse protocols were performed using consecutive 100 ms step changes from −160 to +60 mV with increments of 20 mV. Oocytes were clamped at a holding potential of 0 mV. Data were sampled at 5–10 kHz and filtered at 1–5 kHz. Error bars correspond to the mean ± SEM from a number (n) of independent experimental observations. ANOVA and student t-tests were used to test statistical significance (p<0.05).

## Results

### GIRK5 Localizes to the Nucleus and the Endoplasmic Reticulum within the Animal Pole of *Xl* Oocytes

**Figure 2:**
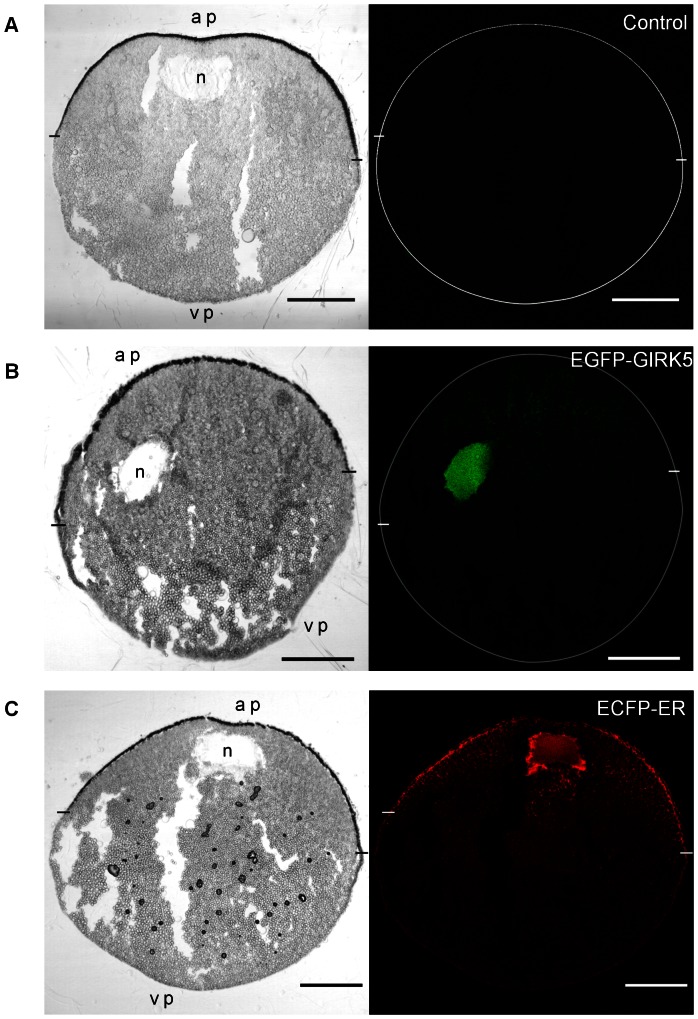
Localization of GIRK5 in an *Xl* oocyte. Images on the left and right show light transmission and confocal images, respectively. The animal pole (ap) and vegetal pole (vp) are shown at the top and the bottom of each panel, respectively. Oocyte circumference (A and B) and the limits between the animal and vegetal poles are indicated on confocal images with a white circle and dashes, respectively. The nucleus (n) is shown on the light transmission images. A) Non injected; B) Injected with EGFP-GIRK5, which localized in the nucleus at the animal pole (green); C) Injected with ECFP-ER, which labeled the ER (red). Scale bar: 250 µm.

**Figure 3:**
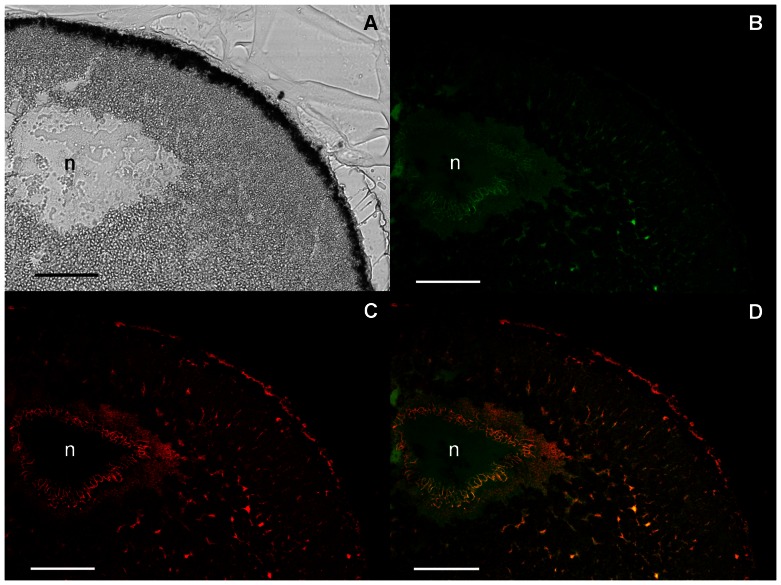
Polarization of GIRK5 within the animal pole. **A**) Light transmission image of the oocyte animal pole section; B,C,D) Confocal microscopic image of the same animal pole section visualizing EGFP-GIRK5 (B, green), ECFP-ER (C, red) or both (D). GIRK5 localized both in the nucleus (n, green) and the ER (yellow). A 40× objective was used. Scale bar: 100 µm.

In order to determine the localization of GIRK5 in *Xl* oocytes, we initially used confocal microscopy to follow two constructs: an endoplasmic reticulum (ER)-enhanced cyan fluorescent protein marker (ECFP-ER) and EGFP-GIRK5. ECFP-ER was observed from the nuclear membrane throughout the cytoplasm, mostly within the animal pole (Figs. 2C and 3C), up to the plasma membrane, accordingly to previous studies of ER distribution in immature oocytes [24]. The EGFP-GIRK5 construct was also localized in the perinuclear space and the ER within the animal pole, but additionally, it was detected in the nucleus (Figs. 2B and 3B). This was more clearly seen after the co-injection of EGFP-GIRK5 and ECFP-ER mRNAs, where the perinuclear space and ER appeared yellow from the co-localization of both markers, but the nucleus appeared green from the sole presence of EGFP-GIRK5 (Fig. 3D).

### The Asymmetric Localization of the phospho-null GIRK5 is Dictated by its N-terminus

**Figure 4:**
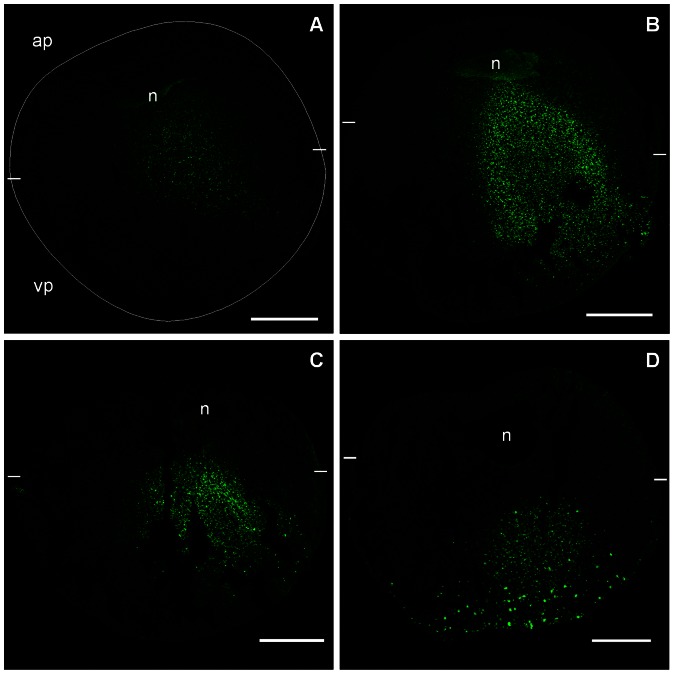
Time-course of GIRK5-Y/A expression. The expression of the EGFP-Y/A construct was observed at different time points after mRNA injection: A) 24 h, a faint expression of Y/A was observed; B) 48 h, Y/A was localized next to the nucleus and in the cytoplasm; C) 72 h, Y/A was in the cytoplasm towards the vegetal pole; D) 96 h, Y/A was in the vegetal pole. The animal pole (ap) and vegetal pole (vp) are shown at the top and the bottom of each panel, respectively. The oocyte circumference is indicated with a white circle in panel A. The limits between the animal and vegetal poles are indicated with white dashes. Scale bar: 250 µm.

Since we previously determined that Y16 is a key residue that determines if GIRK5 is transported to the plasma membrane [15], we investigated how the localization of the phospho-null GIRK5 (EGFP-Y/A) changed over the course of six days. The first day after injection, a faint expression was observed across the animal pole (Fig. 4A). The second and third day, the channel was in the cytoplasm across both poles (Fig. 4 B–C). The fourth day, EGFP-Y/A appeared to have migrated to the vegetal pole displaying a clear punctuate distribution (Fig. 4D). This last localization was maintained until the sixth day (data no shown).

**Figure 5:**
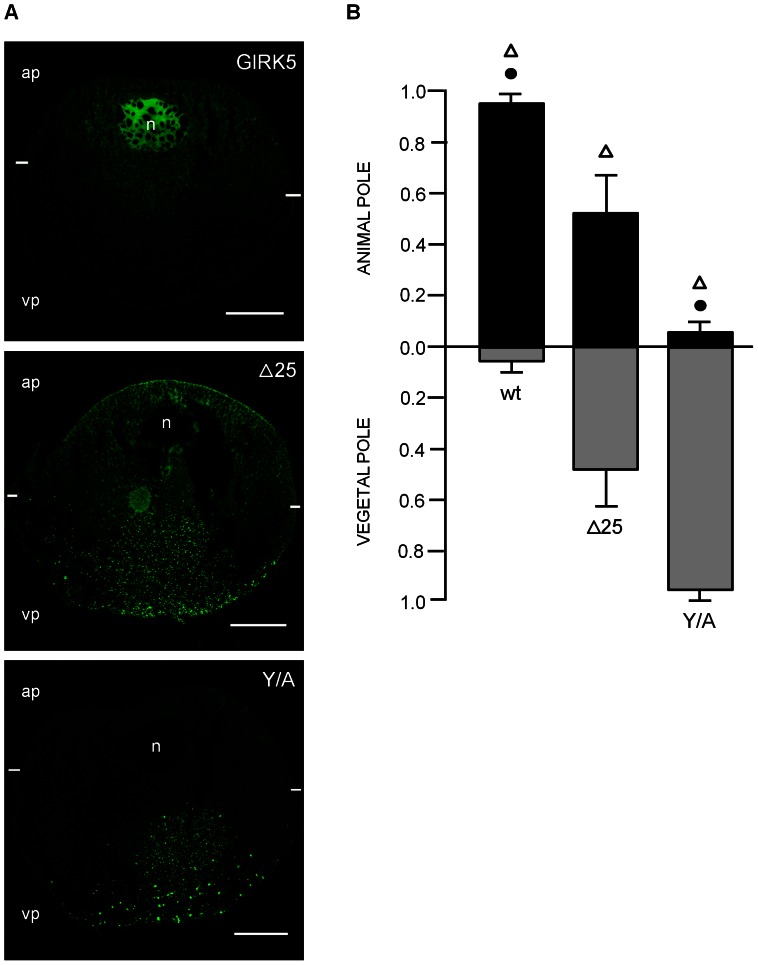
Localization of GIRK5-WT, GIRK5-Δ25 and GIRK5-Y/A. A) Confocal microscopy images of EGFP chimeras of GIRK5, GIRK5-Δ25 and GIRK5-Y/A. B) Fluorescence quantification. GIRK5 was localized at the animal pole (*P*<0.001); GIRK5-Δ25 showed an even distribution in the whole oocyte (*P*>0.001); GIRK5-Y/A localized to the vegetal pole (*P*<0.001. Scale bar: 250 µm. Error bars correspond to mean ± SD; n = 4–6. Circle indicates significant differences of the animal pole compared to vegetal pole (*P*<0.001; paired Student’s t test). Triangle indicates that there is a significant difference among them (*P*<0.005; One Way – ANOVA). Auto-fluorescence of water-injected oocytes (control) was subtracted from mutants.

Next, we performed a more quantitative comparison of the distribution pattern of the three EGFP-constructs (GIRK5, Δ25 and Y/A) by averaging the ROI of the animal versus the vegetal pole (Fig. 5B). Clearly the three constructs showed distinct distributions, with GIRK5 mostly localized at the animal pole, Δ25 equally dispersed in the whole oocyte, and Y/A localized at the vegetal pole (Fig. 5A, B). These results confirm the existence of a polarization-sorting motif located within the N-terminus of GIRK5.

### An Acidic Di-leucine Motif is the Sorting Signal

**Figure 6:**
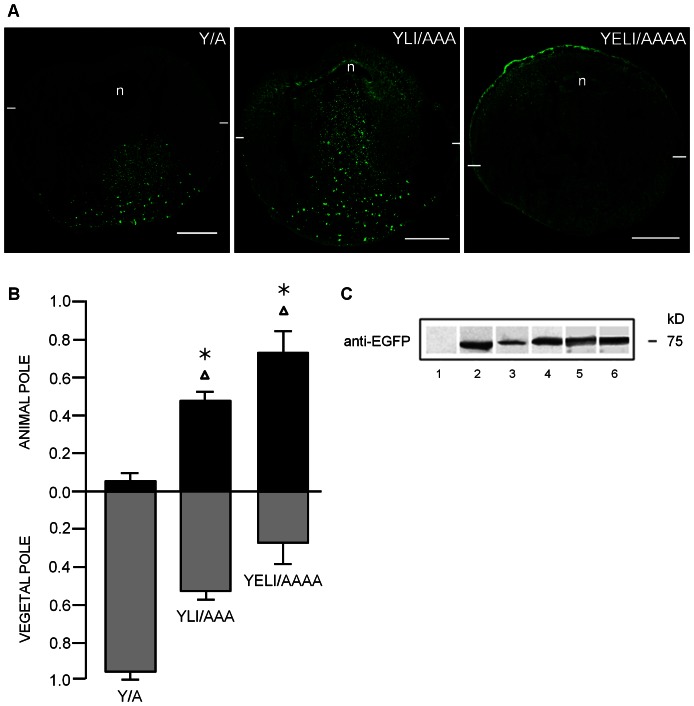
Role of the di-leucine motif in the localization of GIRK5. A) Confocal microscopy images of EGFP chimeras. Removal of the hydrophobic leucine and isoleucine residues in the nonphosphorylated GIRK5 (YLI/AAA), targeted the channel equally to both poles. Remarkably, alanine mutation of the whole di-leucine sorting signal (YELI/AAAA) produced GIRK5 polarization to the animal pole. Scale bar: 250 µm. B) Fluorescence quantification. Error bars correspond to mean ± SD, n = 4–6. Triangle indicates significant differences of oocytes compared to Y16A (*P*<0.001; One-Way ANOVA). Asterisk indicates significant differences of oocytes compared between them (*P*>0.001; One-Way ANOVA). The statistic significance between samples is the same for the animal and vegetal pole. C) Immunoblot analysis revealed bands that correspond to the expected weight of 75 kDa of the EGFP-GIRK5 constructs: 1) Non-injected, 2) GIRK5-Δ25, 3) GIRK5-WT, 4) GIRK5-Y/A, 5) GIRK5-YLI/AAA and 6) GIRK5-YELI/AAAA.

Interestingly, the ESPQLI sequence upstream of Y16 corresponds to an acidic [DE]XXXL[LI] di-leucine motif (Fig. 1) [23]. Therefore, we hypothesized that it could be involved in determining how GIRK5 is trafficked. To test this, we first substituted the key residues within this sequence of the phospho-null EGFP-Y/A to an alanine (Fig. 6A). Quantitative comparison among the different EGFP chimeras showed that the triple mutant YLI/AAA was not polarized (Fig. 6B), but disruption of the acidic motif EXXXLI, corresponding to YELI/AAAA, promoted expression predominantly to the plasma membrane of the animal pole (Fig. 6B). These data thus confirm that the acidic di-leucine motif is important in GIRK5 localization. Immunoblotting of the EGFP-GIRK5 constructs confirmed their expression and the expected size of 75 kDa (Fig. 6C).

### Y16 and I22 have a Dominant Effect in the YESPQLI Sequence

**Figure 7:**
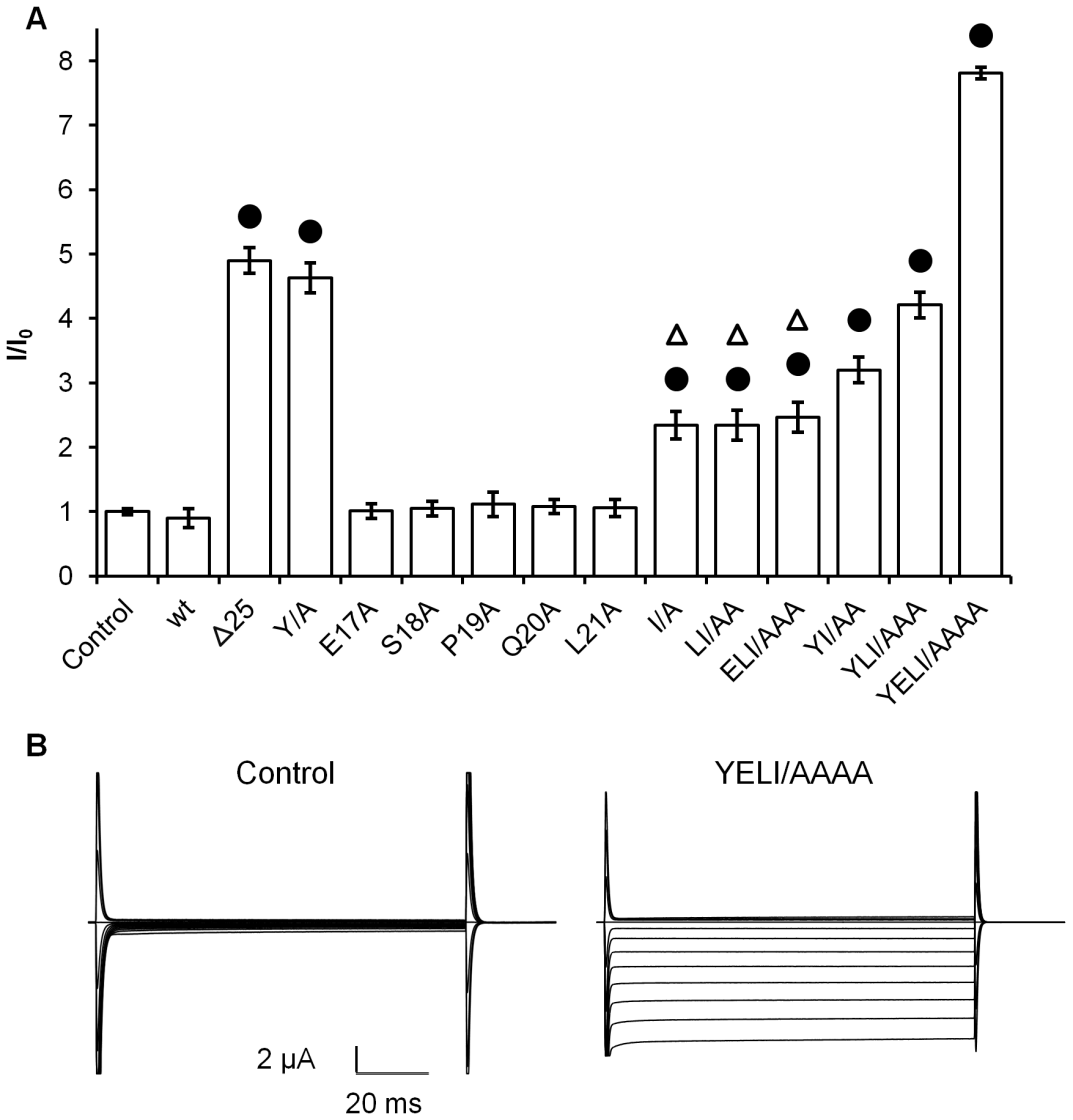
Activity of GIRK5 mutants. A) Normalized currents (I/I0) registered at −160 mV in oocytes injected with H_2_O (control), and mRNA of GIRK5 constructs. Error bars correspond to mean ± SEM from a number (n = 10) of independent experimental observations. A circle indicates a significant difference compared to the control oocytes (*P*<0.01; Student’s *t* test). A triangle indicates no significant difference between oocytes (*P*>0.01; One-Way ANOVA). Normalized currents were registered at −160 mV, (*P*<0.01 for all bars); B) A representative trace recording with control and GIRK5-YELI/AAAA is shown. GIRK5-YELI/AAAA elicited longer potassium inward currents (8.2±0.06 µA). Steps of 100 ms from −160 to +60 mV with increments of 20 mV were applied. Oocytes were clamped at a holding potential of 0 mV and registered in a highly concentrated K^+^ solution (118 mM).

Having determined the 16-YESPQLI-22 sequence as the sorting signal motif, and due to the high electrical activity of ion channels, we proceeded to evaluate the functional expression of the channel mutants. We mutated each one of the amino acid residues to an alanine residue to determine their individual role in GIRK5 activity (Fig. 7A). GIRK5, GIRK5-E17A, GIRK5-S18A, GIRK5-P19A, GIRK5-Q20A and GIRK5-L21A were not functional. However, GIRK5-I22A was functional even when Y16 was not replaced by an alanine residue. Electrical activity was additive only for some mutants: I/A = LI/AA = ELI/AAA<YI/AA<YLI/AAA = Δ25 = Y16A<YELI/AAAA (Fig. 7A) and disruption of the whole acidic di-leucine motif in the phospho-null channel promoted the highest activity (Fig. 7A, B).

### I22 Contributes to GIRK5 Intracellular Retention and the Acidic Residue E20 to the Asymmetric Trafficking

**Figure 8:**
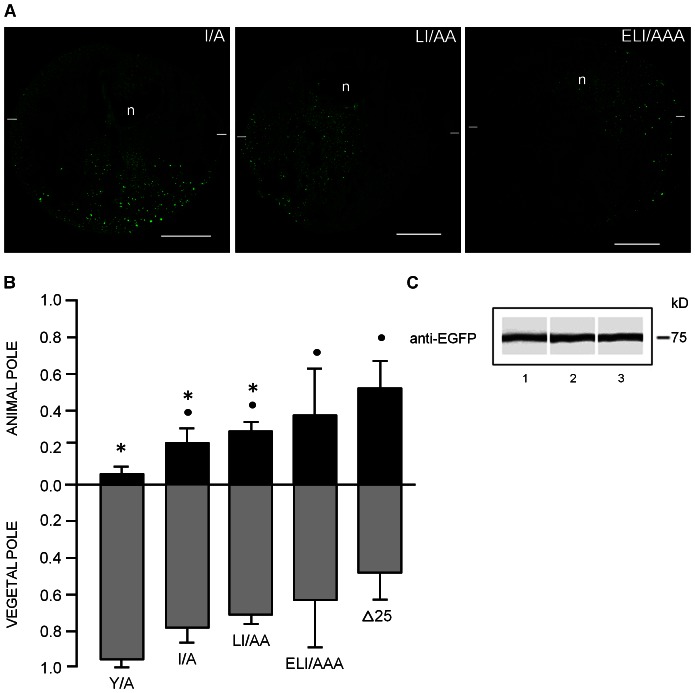
Retention and polarization of GIRK5 mutants bearing the I/A mutation. A) Confocal microscopy assays and B) Quantification of fluorescence show that removal I22 residue in GIRK5, as shown in I/A and LI/AA, causes loss of polarization, whereas the ELI/AAA variant is targeted to both poles like Δ25. Scale bar: 250 µm. Error bars correspond to mean ± SD, n = 4–6. A circle and an asterisk indicate significant differences compared to oocytes expressing Y/A and Δ25, respectively (*P*<0.05; One-Way ANOVA). The statistic significance between samples is the same for the animal and vegetal pole. C*)* Immunoblot analysis of EGFP-GIRK5 mutants with bands corresponding to the expected weight of 75 kDa: 1) I/A, 2) LI/AA, 3) ELI/AAA.

Since I22A was functional even when Y16 was present, we proceeded to analyze the localization of the I/A, LI/AA and ELI/AAA EGFP-constructs. I/A and LI/AA lost ER retention and traveled to the vegetal hemisphere; in contrast, ELI/AAA was distributed across the oocyte as GIRK5-Δ25 (Fig. 8A, B). Therefore, the hydrophobic I22 contributes mostly to GIRK5 intracellular retention whereas glutamate E20 to the asymmetric trafficking.

## Discussion

*Xl* oocytes are fascinating large cells that have been a good model to understand the cell cycle and are the most widely used heterologous expression system. Despite extensive work on *Xl* oocytes, the subcellular distribution of endogenous ion channels remained unknown. In this work, we showed that the important ion channel GIRK5 was retained in the nucleus and the ER at the animal pole (Fig. 3). The asymmetric distribution of GIRK5 might have important repercussions to oocyte development.

During oogenesis, oocyte development has been shown to be greatly affected by asymmetric distribution of mRNAs [3]. For example, some important cytosolic proteins localize to the vegetal cortex. Case in point is the homologue of disheveled (Xdsh), which required for Wnt signaling during the secondary oocyte axis formation [25], [26]; other cases include the proteins X-Stau1 and X-Stau2, which are involved in targeting and anchoring of maternal mRNAs, critical for cell fate determination, to the vegetal cortex of the oocyte [27]; and Fatvg, which participate in cortical rotation and the establishment of the dorsal-ventral body axis in embryos [28]. In the case of GIRK5, it is still unknown what role it plays in oocyte development. Activity of GIRK5 depends of Gβγ dimers. The endogenous pool of these protein dimers maintained oocytes arrested in prophase of meiosis I and sequestration of G-βγ promotes the germinal vesicle breakdown (GVBD) [29]. We thus hypothesize that GIRK5 functional expression would favor this sequestration and therefore, the GVBD.

Asymmetry has been electrically recorded only for a few ion channels. The endogenous nicotinic acetylcholine receptor channel predominates in the vegetal pole [30]; in contrast, calcium-dependent chloride channels [31] and exogenously expressed GABA receptors and sodium-voltage gated channels [32] are more abundant in the animal pole. With respect to the spatial distribution of ion transporters, the endogenous Na^+^/K^+^ ATPase has been found in the animal pole [33].

We showed that the distribution of GIRK5 is greatly controlled by phosphorylation at residue Y16. Indeed, a variant of GIRK5 with an N-terminal deletion (Δ25) or bearing a mutation at residue Y16 (Y/A) showed completely different distributions. The former localized across the entire oocyte, whereas the latter showed mostly at the animal pole (Fig. 5). This implicated that there were more residues at the N-terminus involved in the localization of GIRK5. As we demonstrated later, the acidic di-leucine motif adjacent to Y16 (sequence ESPQLI) also has an important role in GIRK5 distribution.

Di-leucine motifs are defined as [DE]XXXL[LI]. Besides the obvious importance of the Leu residues, it is known that the nature and position of the XXX residues within the motif are important [17], [34]. For example, the glucose transporters GLUT8 and GLUT12 both have a di-leucine motif but with different sequences. GLUT8 has a XXP sequence that directs it to lysosomes. It is then not surprising that GIRK5, which has an XPX sequence, shares the same fate as GLUT12 and it is directed to the plasma membrane. Other examples of membrane proteins that rely on di-leucine motifs for trafficking include NPP1, an enzyme of the nucleotide pyrophosphatase/phosphodiesterase family [35], the chlorine channel CIC-2 [36], and rat GIRK2, which besides the di-leucine motif it contains a close phosphorylable Ser residue that functions as a switch that regulates its surface expression [37].

Similar to rat GIRK2, the localization and localization of GIRK5 depends both on its phosphorylable Y16 and the key di-leucine residue I22. This was demonstrated not only by different distributions of Y/A and I/A variants, but also by measuring their electrical activity (Fig. 7), which suggested that residues near or in the acidic di-leucine motif contribute to the proper recognition of the N-terminus for ER retention. This is somewhat surprising as usually di-leucine motifs are forward ER trafficking signals of G-protein coupled receptors [38]–[44]. However, the di-leucine motif of the synaptic adhesion-like molecule 1 (SALM1) also functions as ER retention signal [45]. It will be interesting to see if future studies reveal the mechanisms dictating such distinct ER trafficking responses to di-leucine motifs.

In conclusion, GIRK5 is polarized to the vegetal pole of *Xl* oocytes thanks to its phosphorylable Y16 residue and its adjacent acidic di-leucine motif. These findings represent an important stepping stone in understanding how ion channels are transported in the maturation and fertilization model of *Xl* oocytes. We intend to carry out similar experiments of other membrane proteins of this important cellular system in the future.
